# A novel preference-informed complementary trial (PICT) design for clinical trial research influenced by strong patient preferences

**DOI:** 10.1186/s13063-021-05164-1

**Published:** 2021-03-12

**Authors:** Samina Ali, Gareth Hopkin, Naveen Poonai, Lawrence Richer, Maryna Yaskina, Anna Heath, Terry Paul Klassen, Chris McCabe, on behalf of the KidsCAN PERC Innovative Pediatric Clinical Trials No OUCH Study Group, Amy Drendel, Gareth Hopkin, Jeff Round, Martin Offringa, Petros Pechlivanoglou, Eleanor Pullenayegum, David Rios, Marie-Christine Auclair, Kelly Kim, Lise Bourrier, Lauren Dawson, Kamary Coriolano DaSilva, Pamela Marples, Rick Watts, Jennifer Thull-Freedman, Patrick McGrath, Timothy A. D. Graham, Lisa Hartling, Tannis Erickson, Brendon Foot, Kurt Schreiner, Julie Leung, the KidsCAN PERC Innovative Pediatric Clinical Trials Methods Core, Martin Offringa, Petros Pechlivanoglou, Eleanor Pullenayegum, Juan David Rios, Jeff Round

**Affiliations:** 1 Department of Pediatrics, University of Alberta, AB Edmonton, Canada; 2 Women and Children’s Health Research Institute, University of Alberta, Edmonton, Alberta Canada; 3 Institute of Health Economics, Edmonton, Alberta Canada; 4 Departments of Pediatrics and Internal Medicine, Schulich School of Medicine & Dentistry, Childrens’ Health Research Institute, London, Ontario Canada; 5 The Hospital for Sick Children, Toronto, Ontario Canada; 6 University of Toronto, Toronto, Ontario Canada; 7 University College London, London, UK; 8 Max Rady College of Medicine, Pediatrics and Child Health, Rady Faculty of Health Sciences, University of Manitoba, Winnipeg, Manitoba Canada; 9 Children’s Hospital Research Institute of Manitoba, Winnipeg, Manitoba Canada

**Keywords:** Clinical trial, Patient preference, Methodology, Caregiver preference

## Abstract

**Background:**

Patients and their families often have preferences for medical care that relate to wider considerations beyond the clinical effectiveness of the proposed interventions. Traditionally, these preferences have not been adequately considered in research. Research questions where patients and families have strong preferences may not be appropriate for traditional randomized controlled trials (RCTs) due to threats to internal and external validity, as there may be high levels of drop-out and non-adherence or recruitment of a sample that is not representative of the treatment population. Several preference-informed designs have been developed to address problems with traditional RCTs, but these designs have their own limitations and may not be suitable for many research questions where strong preferences and opinions are present.

**Methods:**

In this paper, we propose a novel and innovative preference-informed complementary trial (PICT) design which addresses key weaknesses with both traditional RCTs and available preference-informed designs. In the PICT design, complementary trials would be operated within a single study, and patients and/or families would be given the opportunity to choose between a trial with all treatment options available and a trial with treatment options that exclude the option which is subject to strong preferences. This approach would allow those with strong preferences to take part in research and would improve external validity through recruiting more representative populations and internal validity. Here we discuss the strengths and limitations of the PICT design and considerations for analysis and present a motivating example for the design based on the use of opioids for pain management for children with musculoskeletal injuries.

**Conclusions:**

PICTs provide a novel and innovative design for clinical trials with more than two arms, which can address problems with existing preference-informed trial designs and enhance the ability of researchers to reflect shared decision-making in research as well as improving the validity of trials of topics with strong preferences.

## Background

Patients and families are often presented with treatment options that require them to consider the efficacy of treatments, their personal preferences, and the values and weight they place on health outcomes [1]. In clinical practice, patients, families, and clinicians can work towards shared decision-making that achieves a balance between effectiveness and preferences [2]. However, in research, patients and families must choose whether to give consent and receive an allocated treatment or be excluded. This can pose problems for research both ethically (i.e., shared decision-making) and practically (i.e., study feasibility, validity).

Preferences in healthcare arise for a number of reasons and reflect patients’ and families’ personal values regarding treatment options. When they are strongly held, preferences may drive treatment decisions regardless of the effectiveness of treatments and consideration of preferences is crucial to effective shared decision-making [1]. Awareness of patient preferences and their importance has increased over recent years, but there is often still a mismatch between what health professionals believe patients value and what they actually value [3]. In research, there is still limited focus on patient preferences and novel approaches are needed to ensure that preferences are considered when planning studies.

### Preference-informed trial designs

Randomized controlled trials (RCTs) are considered the gold standard of effectiveness research and are the design of choice for comparing interventions in healthcare [4]. However, when patients have strong preferences that directly influence treatment decisions, the traditional RCT design may be problematic. The *external* validity of trials relies on recruiting a sample which is representative of the patient group as a whole. Where strong preferences are present, traditional RCTs may be unable to recruit patients who would not accept random allocation to an intervention, and this may lead to unrepresentative samples or could undermine trial feasibility. Additionally, the *internal* validity of trials relies on randomized patients having similar adherence to treatment options and retaining patients for the duration of follow-up in the trial. Where strong preferences are present, patients may drop out or cross-over if they receive an intervention they do not want or feel uncomfortable about being blinded.

Some attempts to adapt the RCT design have been made [5] with preferences recorded after consent but prior to randomization and this information being used in analyses to explore whether treatment effects differ according to preference. When preferences are not strongly held, this approach has been acceptable [6], but where preferences are more strongly held, innovative trial methods are needed to ensure representative samples are recruited and retained. Partially randomized preference trials and doubly randomized preference trials have both been developed to incorporate consideration of preferences, but for both designs, there are outstanding issues [6, 7].

In partially randomized preference trials, patients who wish not to be consented to randomization are able to opt for their preferred treatment while patients without strong preferences are randomized using traditional methods [6, 8]. This approach addresses some concerns regarding external validity as patients with strong preferences are still able to take part in the research. It also may help with issues of internal validity if participants have higher levels of adherence and can be retained in greater numbers at follow-up. However, this method introduces biases that may limit its use. Patients who choose to select their treatment rather than being randomized become an observational cohort with the potential for high levels of selection bias. This limits the validity of including these participants in pooled analyses with randomized participants, and this analytic approach cannot address issues of bias. Furthermore, there are practical challenges in using this approach with some research questions. If only one of multiple treatment options is subject to strong preferences, there is the possibility that only a small number of patients would opt for randomization or for one of the treatments. This would lead to the loss of the randomization element of the trials and introduce an imbalance across preference arms leaving it of little value.

A recently published partially randomized preference trial adopted this approach after initial problems with recruiting for a traditional RCT [9]. Recruitment rates increased after investigators allowed participants to choose either traditional randomization or their preferred treatment (i.e., ambulatory or inpatient care) for severe nausea and vomiting of pregnancy. Data from both randomized and preferred treatment arms were pooled for analysis, but this approach seems undermined by a difference between the characteristics of the groups at baseline. For the primary outcome, those choosing inpatient treatment had the highest nausea/vomiting score in the four groups, and those choosing the outpatient treatment had the lowest score. The issues presented by these differences and their impact on the results were not fully explored. Indeed, the use of a partially randomized preference trial design improved recruitment rates and external validity, but worsened threats to internal validity.

Another option for preference-informed trials is the doubly randomized preference trial design. In this design, patients are randomized either to a random group with conventional randomization to intervention arms or to a choice group where they are able to choose their intervention [7]. This approach may help to address issues of internal validity as participants in the random and choice groups should be similar, but nonetheless issues of selection bias may remain within the choice group [10]. The doubly randomized preference trial design may increase the number of patients who choose to consent as there is a lower chance that they may be ultimately randomized to a less preferred treatment. However, in research questions with strong preferences, a number of patients may still be unwilling to consent to this. This can translate to problems with external validity that are also seen in traditional RCTs.

In an example of a doubly randomized preference trial, Zoellner et al. [11] examined the comparative effectiveness of prolonged exposure to triggers of traumatic stress compared to sertraline for the treatment of post-traumatic stress disorder and randomly allocated participants to a random assignment group or a choice group. From the outset, they expected a preference for prolonged exposure and high levels of drop-out and non-adherence in the sertraline group. These expectations were borne out, and threats to internal validity were introduced with many more participants in the choice arm selecting prolonged exposure than sertraline. In addition, there were low levels of adherence and high levels of drop-out, particularly for the randomized to sertraline groups. From the trial reporting, it is difficult to ascertain the proportion of eligible participants who gave consent, but it seems plausible that those with the strongest aversion to medication and/or desire for prolonged exposure would not be included in the study, raising similar issues of external validity that one might expect with a traditional randomized clinical trial.

Both of these preference-informed trial approaches are useful in some circumstances, but there continues to be a need for novel innovative designs for research questions where there are strongly held preferences which will have a large impact on the behavior of eligible patients. In this paper, we propose a novel and innovative design for comparative effectiveness trials which include three treatment options and a patient group who may have a strong aversion to one of the treatment options. This innovative design for clinical trials with more than two arms was motivated by a comparative effectiveness trial of pain medications in the emergency department for pediatric musculoskeletal (MSK) injury where families often have a strong preference to avoid opioids [12, 13]. This design could improve both *internal* and *external* validity compared to traditional randomized controlled trials and existing preference-informed trial designs. In addition, it would respect values of shared decision-making and patient and caregiver autonomy in the research setting and could give real-world insights into the impact of preference on treatment decisions.

## A novel preference-informed complementary trial (PICT) methods

### Design

The preference-informed complementary trial (PICT) design is a method that has been designed to allow patient and caregivers’ preferences to be reflected within the research context and to address the methodological limitations of other preference-informed designs. The design relies on two complementary trials being implemented within a single study and allows patients/caregivers to select between trials at the same entry point. The complementary trials are designed as collaborative trials and would function in parallel to avoid duplication of effort and resources that would be needed to provide the trials separately.

**Fig. 1:**
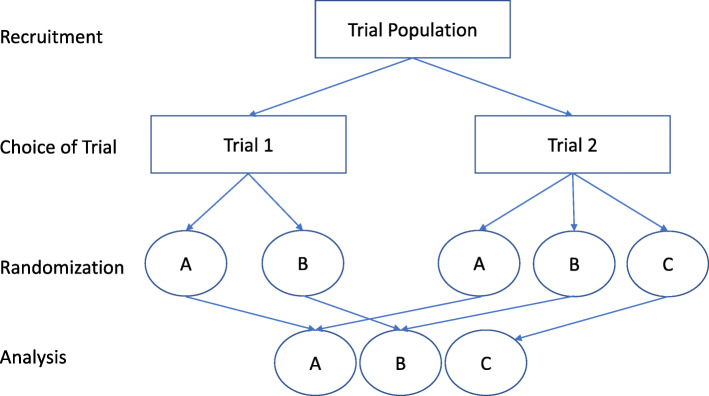
Schema of the PICT design using a pooled analysis. Participants would be asked to choose between two trials with Trial 2 containing all three treatment options (A, B, C) and Trial 1 containing two treatment options (A, B) and omitting the option with strong preferences that may deter participation (C). Participants would then be randomized to the available treatment options according to their choice of trial. If there is no clinical or statistical evidence that trial populations have important differences, then data from all arms can be pooled for a single analysis

In the PICT design, patients and families are asked whether they would prefer to participate in one of two simultaneously occurring complementary, or ‘sister,’ trials (see Fig. 1). These two trials contain differing numbers of interventions, with one trial including all possible interventions and one trial excluding the intervention that is subject to strong preferences, allowing patients and families to be included in research but to avoid a specific treatment option. After patients and families have chosen the trial based on their preference, they are randomized to the interventions included in the trial of their choice. Trial procedures from traditional randomized controlled trials are then followed, with the two complementary trials sharing identical procedures, and running in parallel, until the completion of both trials. The use of two complementary trials allows those who have a strong preference to avoid a particular treatment option while still being randomized to the other available treatment arms. It is envisaged that this approach could lead to all patients being pooled in a single analysis, if statistically and clinically appropriate, which would lead to increased power and improved generalizability.

### Analysis

The populations in the complementary trials may be different, as they do not have the same preferences. If these preferences do NOT influence the treatment effect, then the two trials could be analyzed together to estimate the single common treatment effect. This would improve the power of the study and our ability to generalize study results to different patient populations. In PICT trials, a family’s expression of preference is, by definition, measured at baseline (i.e., at the time of recruitment). Randomization should, in theory, allow for equal distribution of all other unknown variables between the populations, so long as they are not conditional on preference, as preference of trial choice is the only factor that influences the probability of receiving any one of the treatments at the time of randomization. However, to erroneously estimate a single treatment effect would lead to biased, potentially imprecise, and thus misleading results. To mitigate this potential risk, we propose that a single treatment effect should be estimated within PICTs unless there is clinical or statistical evidence of an important, fundamental difference between the two populations, with a separate treatment effect estimated for each trial as a sensitivity analysis.

We suggest that the clinical rationale for estimating a single treatment effect should focus on whether the baseline characteristics across the complementary trials are similar based on clinical meaningfulness and acceptability to knowledge users. In order to determine this, we will engage clinicians to indicate the level of differences in baseline characteristics of the two complementary trials that would be seen as acceptable for estimating a single treatment effect. To minimize the bias in this decision-making, pre-specified decision rules should determine a maximum tolerated difference between the trials for each of the baseline characteristics and these decisions should be guided by feasibility and pilot studies. It is possible that several patient characteristics could strongly influence the preference for one treatment over another; however, if there is clinical rationale that these cannot be effect modifiers, then they can be excluded. If a clinical rationale for estimating a single treatment effect has been established, we suggest a formal statistical testing procedure to determine whether there is statistical evidence that the complementary trials should be analyzed separately. This statistical testing should assess whether there is a statistically significant difference between the treatment effects for the primary outcome across the trials. Only if there is no evidence, statistically or clinically, for a different treatment effect across trials should a single treatment effect estimate be provided. By including a statistical and clinical testing procedure, we will ensure that the analysis of the PICT will be acceptable within the clinical community, even in the settings where the test of significance for the trial by treatment interaction is underpowered.

The analysis plan for PICTs should include details on both the clinical and statistical testing procedures that will be used. It is advisable that the analysis plan outlines the analytical procedures that would be followed in both cases, with and without a single treatment effect. Separate analysis of the two trials should also be included as a supplementary analysis, if the primary analysis focuses on estimating a single treatment effect.

### Sample size considerations

As the analysis for PICTs may require a separate analysis for each trial, we suggest that they are powered separately. This means that the sample size requirements for PICTs are larger than a standard RCT. We also recommend that the recruitment rates into each trial are monitored closely, with potential adjustments made if required to ensure the feasibility of the trials, i.e., stopping a trial early or changing randomization ratios to ensure enough patients receive the intervention that is subject to strong preferences. The impact of these adjustments on the statistical power of the individual trials should be assessed and reported clearly.

## Discussion

### Motivating example

Our research team wished to examine pain management options for children with MSK injury [14–16]. Ibuprofen has been established as first-line therapy for acute MSK pain, but is often inadequate in isolation [15, 17, 18]. As such, there was a need to identify an adjuvant medication to augment the analgesia provided by ibuprofen. An opioid, oral hydromorphone, has been shown to be effective in other contexts [19, 20] and is appropriate as a candidate for comparison to other non-opioid analgesic options (ibuprofen; ibuprofen + acetaminophen). The addition of acetaminophen to ibuprofen was included because it has been shown to be effective for other patient groups [19, 20] and, if shown to have a similar additive efficacy in this setting, would be a non-opioid option. Despite the known effectiveness of opioids for moderate to severe pain [21], the opioid crisis in North America means that many patients and families may have strong preferences to avoid opioids. Deaths from opioids in the USA and Canada have reached epidemic levels [22, 23], and media, third sector, and health service responses have increased the salience of the dangers of non-medical use of opioids [24]. Due to this, along with the unknown risk of developing opioid use disorders after short-term therapeutic use, families may want to avoid exposure to opioids in a clinical trial regardless of their potential effectiveness; recent empirical work supports this notion and has suggested that less than 50% of caregivers would accept opioids for moderate pain after MSK injury [13].

Due to this opioid reluctance, it was decided that a traditional RCT and a doubly randomized preference trial would have low recruitment rates. Similarly, a partially randomized controlled trial may have limited recruitment to the randomized group and an imbalance between caregivers choosing against oral hydromorphone. Due to these limitations, the PICT approach was conceived for the Non-Steroidal or Opioid Analgesia Use for Children with Musculoskeletal Injuries (No OUCH) study which began recruitment in early 2019 (ClinicalTrials.gov Identifier: NCT03767933) [25].

In the No OUCH PICT, caregivers are asked whether they would like to participate in a 3-arm trial which includes all possible treatment options (oral ibuprofen + oral hydromorphone; oral ibuprofen alone; oral ibuprofen + oral acetaminophen) or a 2-arm trial arm which does not include the opioid treatment (oral ibuprofen alone; oral ibuprofen + oral acetaminophen). This approach allows caregivers who would be reluctant to take part in the trial if there was a chance of receiving opioids to be included, allowing caregivers’ opioid preferences to be reflected in the research design.

Our motivating example will also embed a preference survey into the study to examine whether families’ characteristics, prior experience with opioid treatment or substance misuse, or source of injury differ according to preference. A qualitative sub-study will also use interviews to further understand caregiver decision-making.

To determine whether the two trials have clinically different baseline characteristics, we will evaluate these characteristics at the interim analysis at the mid-point of the No OUCH trials. We will tabulate these baseline characteristics as though they are to be included in the final report. We will then ask a group of experts whether they think the results of a trial in one of these populations are relevant to the other. If they do not believe the two populations are similar, based on this interim analysis, then a discrete choice experiment will be undertaken within the Pediatric Emergency Research Canada network to determine a formal rule for pooling. Statistical testing for different treatment effects across the two trials will be undertaken using nested linear mixed models. Specifically, the full model will include a treatment by trial interaction term while the reduced model will only include two treatment effect parameters. A likelihood ratio test will be used to declare whether the treatment by trial interaction is significant.

**Fig. 2:**
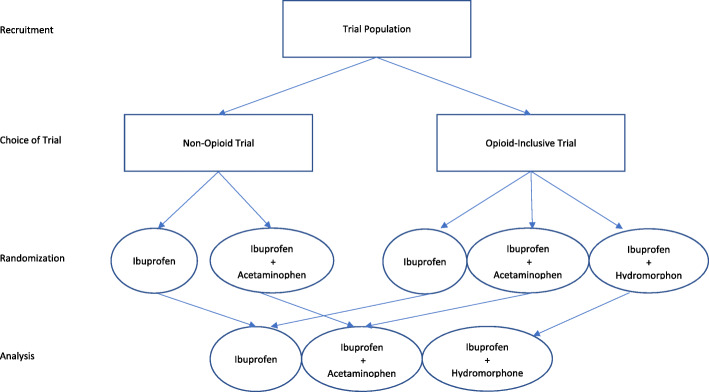
Schema of the No OUCH trials using a pooled analysis. Participants would be asked to choose between two trials with the opioid-inclusive trial containing all three treatment options (ibuprofen; ibuprofen + acetaminophen; ibuprofen + hydromorphone) and non-opioid trial containing non-opioid options (ibuprofen; ibuprofen + acetaminophen) and omitting the opioid option with strong preferences that may deter participation (ibuprofen + hydromorphone). Participants would then be randomized to the available treatment options according to their choice of trial. If there is no clinical or statistical evidence that trial populations have important differences, then data from all arms can be pooled for a single analysis

**Fig. 3:**
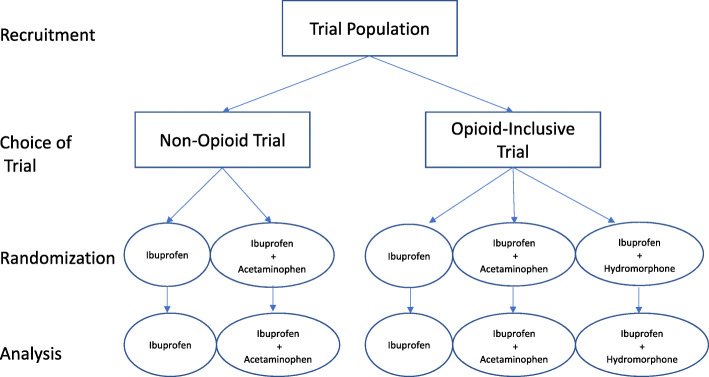
Schema of the No OUCH trials using a separate analysis. Participants would follow the procedure described in Fig. 2, but if important clinical and statistical differences were found between trial populations which prevented pooling, then they would be analyzed separately and alternative methods for synthesizing the results of the trial would be used

Both trials have a power of 90% to detect the treatment effect, with sample sizes of 170 and 315 for the two- and three-arm trials, respectively, giving a power for the combined analysis of 98%. Thus, the minimum sample size of the No OUCH PICTs is 1.54 times higher than the equivalent three-arm trial. The No OUCH trials also include an interim assessment of the recruitment rates into the two trials. Thus, the sample size may change as we could increase the proportion of patients randomized to oral ibuprofen + oral hydromorphone in the three-arm trial if there is a high imbalance in recruitment rates, provided that the power of the three-arm trial remains above 80% [26]. If the treatment by trial interaction is not significant, then the trials will also be analyzed separately as sensitivity analyses (Fig. 2). If there is clinical or statistical evidence that the treatment effect is not the same across the two trials, indicating confounding or effect modification, then the trials will re-analyzed and reported separately (Fig. 3). In both eventualities, the PICT approach is superior to traditional RCTs or other preference-informed trials. On the one hand, if no differences exist, recruitment will have been maximized. On the other hand, if differences do exist, then a group who would have not otherwise been recruited will be included in the study. The complete statistical analysis plan is published and available for reference [26].

### Strengths and limitations of the preference-informed complementary trials design

A key advantage of the PICT design is that it allows patients with a strong preference to avoid a particular treatment and still participate in the research process, thereby increasing the external validity of the trial. In the No OUCH study, it is anticipated that more caregivers will agree to participate in the study than if we ran a single trial with an opioid arm. This will improve the external validity by increasing the number of eligible participants enrolled in the study compared to other trial methodologies.

The PICT design can also improve internal validity by reducing drop-out, poor adherence, and cross-over by allowing patients to avoid treatment options to which they have a strong aversion or to select a trial with more favorable options. These issues would not be seen in the motivating No OUCH trial due to the immediacy of the intervention and short-term outcomes (i.e., oral administration at randomization and change in pain score at 60 min). However, in trials with different treatment modalities (i.e., pharmacologic and psychological arms), this may be particularly important.

Another benefit of the PICT design is that a larger number of eligible patients and families are included in the research. This allows for a quantitative and qualitative exploration, in a more representative sample, of why strong preferences for different treatment options are present. This mixed methods approach could ensure that as much information as possible is learned from trial-based research [27]. In cases where effectiveness may differ according to preferences, this will also provide valuable information to improve understanding of treatment mechanisms and responses in real-world scenarios.

There are threats to internal validity with the PICT design. If preferences are driven by or associated with confounders or effect modifiers, pooled analyses are not appropriate. For example, in the No OUCH trial, if preferences lead to substantial differences in age, pain score at baseline, or type of injury, then pooling would not be appropriate as these variables could be confounders or effect modifiers. This issue also affects partially randomized preference trials. In the PICT design, the population with strongly held preferences will be included in the study and will still be randomized to the treatment options which they are willing to receive. Thus, the PICT design still determines the best treatment option for this group and can guide decisions in the real world where clinicians face questions about the most effective treatment for those with strong treatment preferences.

In the No OUCH study, the two complementary trials are powered to detect a difference within the trial and recruitment will need to continue into both trials until recruitment targets are met. This means that a greater number of participants compared to a traditional 3-arm randomized trial would be needed, with over-recruitment of the 2-arm trial. Thus, a PICT trial may take longer and be more resource intensive than other trial designs. However, the benefits of using the PICT design will often outweigh these concerns and provide more comprehensive analysis across a spectrum of research questions. A PICT design will also guard against loss of validity if a high level of patients decline to be exposed to a particular treatment option. In addition, the cost of recruiting additional participants or delivering additional interventions may be limited compared to the resources needed to set up and deliver a second separate clinical trial of any size. The PICT design is a proposed solution to the problem of patient preferences making a trial unfeasible. Hence, while a larger sample size may extend the recruitment period for a PICT trial, we would only expect this trial design to be employed when a conventional trial design is deemed unfeasible.

## Conclusions

In healthcare, patients and families may have strong preferences about the treatments they receive that go beyond considerations of efficacy. When interventions have such preferences attached, traditional RCTs and preference-informed designs have issues with internal and external validity that need to be addressed. The novel and innovative preference-informed complementary trial (PICT) design can address these issues while also allowing patients’ and families’ values to be reflected and understood in research and ensure trial funding is used efficiently. In our motivating example, a multi-armed trial with opioids in acute pain for MSK injury, the PICT is the preferable design. It allows patient and family preferences to be reflected and understood in research. It also ensures that recruitment is maximized and questions that are relevant to clinical practice can be answered in a timely manner. Many other research questions could benefit from this approach, which addresses issues with both *internal* and *external* validity. Thus, research in areas with strong preferences should consider this design alongside traditional RCTs and other preference-informed designs. The No OUCH trial will provide information on the implementation of PICT trials and will provide guidance to support teams who wish to replicate this approach in future studies.

## Data Availability

Not applicable

## Abbreviations

MSK: Musculoskeletal
No OUCH: Non-Steroidal or Opioid Analgesia Use for Children with Musculoskeletal Injuries
PICT: Preference-informed complementary trial
RCT: Randomized controlled trial
