# Correction to: A novel preference-informed complementary trial (PICT) design for clinical trial research influenced by strong patient preferences

**DOI:** 10.1186/s13063-021-05312-7

**Published:** 2021-05-20

**Authors:** Samina Ali, Gareth Hopkin, Naveen Poonai, Lawrence Richer, Maryna Yaskina, Anna Heath, Terry Paul Klassen, Chris McCabe, on behalf of the KidsCAN PERC Innovative Pediatric Clinical Trials No OUCH Study Group, the KidsCAN PERC Innovative Pediatric Clinical Trials Methods Core

**Affiliations:** 1 Department of Pediatrics, University of Alberta, AB, Edmonton, Canada; 2 Women and Children’s Health Research Institute, University of Alberta, Edmonton, Alberta Canada; 3 Institute of Health Economics, Edmonton, Alberta Canada; 4 Departments of Pediatrics and Internal Medicine, Schulich School of Medicine & Dentistry, Childrens’ Health Research Institute, London, Ontario Canada; 5 The Hospital for Sick Children, Toronto, Ontario Canada; 6 University of Toronto, Toronto, Ontario Canada; 7 University College London, London, UK; 8 Max Rady College of Medicine, Pediatrics and Child Health, Rady Faculty of Health Sciences, University of Manitoba, Winnipeg, Manitoba Canada; 9 Children’s Hospital Research Institute of Manitoba, Winnipeg, Manitoba Canada

**Correction to: Trials 22, 206 (2021)**

**https://doi.org/10.1186/s13063-021-05164-1**

Following the publication of the original article [1], we were notified that the Acknowledgements section needs to accommodate additional collaborators.

The original article has been corrected.
